# Resistance Training Complements Anti-TNF Therapy in DSS-Induced Colitis by Improving Skeletal Muscle Inflammatory and Mitochondrial Gene Signatures

**DOI:** 10.3390/cimb48060568

**Published:** 2026-05-28

**Authors:** Ya Zhu, Xiaochun Zhang, Chao Jin, Heying Jin

**Affiliations:** The Second Clinical Medical College, Nanjing University of Chinese Medicine, Nanjing 210017, China; zhuya202509@163.com (Y.Z.); 202430161@njucm.edu.cn (X.Z.); liujianlei2025@163.com (C.J.)

**Keywords:** anti-TNF-α, inflammatory bowel disease, mitochondria, oxidative phosphorylation, resistance training, transcriptomics

## Abstract

Inflammatory bowel disease (IBD) is associated with skeletal muscle loss and mitochondrial dysfunction, which may compromise therapeutic responsiveness. We investigated whether progressive resistance training could improve muscle oxidative phosphorylation gene expression and enhance the efficacy of anti-TNF-α therapy (infliximab) in a dextran sulfate sodium (DSS)-induced colitis mouse model. Male C57BL/6 mice with subacute DSS-induced colitis were assigned to control, DSS, DSS + resistance training, DSS + anti-TNF-α, and DSS + resistance training + anti-TNF-α groups (*n* = 6 each). Resistance training consisted of 8 weeks of progressive ladder climbing; anti-TNF-α was administered at weeks 0, 2, and 6. Outcomes included body weight, disease activity, muscle function, histology, serum cytokines, muscle transcriptomics, and qPCR. DSS caused weight loss, functional decline, elevated proinflammatory cytokines, suppressed mitochondrial gene expression, and muscle inflammation. Resistance training or anti-TNF-α alone partially restored performance and mitochondrial gene expression while reducing inflammation. Their combination yielded superior effects (*p* < 0.01), normalizing histology. These findings suggest that resistance training may improve muscle metabolic status and attenuate systemic inflammation, thereby contributing to enhanced anti-TNF-α therapy outcomes in this preclinical model. Further studies are warranted to elucidate the underlying mechanisms and evaluate the translational potential.

## 1. Introduction

Inflammatory bowel disease (IBD), encompassing ulcerative colitis (UC) and Crohn’s disease (CD), is a chronic relapsing autoimmune disorder of the gastrointestinal tract. Its global incidence has risen sharply in recent decades, particularly in Asia [1,2,3]. Current management focuses on controlling inflammation and achieving remission through aminosalicylates, corticosteroids, immunosuppressants, and biologic agents [4]. The introduction of infliximab (IFX), an anti-tumor necrosis factor-α (TNF-α) monoclonal antibody, significantly improved clinical outcomes in moderate to severe IBD [5]. However, 30–50% of patients exhibit primary nonresponse or secondary loss of response, driven by immunogenicity, high inflammatory burden, pharmacokinetic variability, and emerging mechanisms such as oncostatin M (OSM)-driven stromal resistance [6,7,8]. Furthermore, disrupted mitochondrial homeostasis correlates closely with anti-TNF refractoriness [9], highlighting the need for strategies that concurrently address immune and metabolic dysfunction in IBD.

Skeletal muscle is increasingly recognized as a critical immunometabolic organ. In IBD, chronic systemic inflammation disrupts muscle protein homeostasis, leading to muscle wasting and functional impairment in 30–52% of patients, which correlates with worse prognosis and reduced quality of life [10,11]. Mechanistically, elevated TNF-α and IL-6 drive NF-κB activation and ubiquitin–proteasome-mediated proteolysis while inhibiting the mTOR pathway, accelerating muscle protein catabolism [12]. Conversely, healthy contracting muscle secretes anti-inflammatory myokines such as IL-10, which suppress systemic inflammation and promote M2 macrophage polarization [13]. In patients with muscle wasting, diminished myokine release further amplifies systemic inflammation, perpetuating a vicious cycle [14]. Malnutrition, malabsorption, and physical inactivity compound these effects by impairing muscle mitochondrial function and oxidative phosphorylation, thereby reducing the body’s resilience against inflammatory insults [15,16]. Interventions that restore muscle metabolic homeostasis may therefore help rebalance immune regulation and improve outcomes of biologic therapy.

Resistance training stimulates both muscle hypertrophy and mitochondrial biogenesis, enhancing oxidative phosphorylation capacity and energy metabolism efficiency [17]. It also shifts the myokine profile toward an anti-inflammatory phenotype [18,19,20]. However, the efficacy and safety of resistance training specifically in IBD are not established. Most evidence derives from healthy or aging populations, and some clinical observations suggest that high-intensity exercise may transiently worsen gastrointestinal symptoms in IBD. The optimal training modality, intensity, and duration for this population remain undefined. Moreover, most mechanistic insights originate from rodent exercise models that differ substantially from voluntary human exercise.

In this context, we hypothesized that progressive resistance training would enhance skeletal muscle mitochondrial gene expression, reduce systemic inflammation, and thereby complement anti-TNF-α therapy in a DSS-induced colitis mouse model. We emphasize that the DSS model does not recapitulate clinical biologic resistance, and that mitochondrial outcomes are confined to transcript-level analyses. By integrating physiological, histological, biochemical, transcriptomic, and targeted gene expression data, this study provides initial preclinical evidence on whether muscle-targeted interventions may contribute to a more favorable therapeutic milieu under anti-TNF-α exposure.

## 2. Materials and Methods

### 2.1. Reagents

The primary reagents used and their sources are as follows: dextran sulfate sodium (DSS; Shanghai Maclin Biochemical Technology Co., Ltd., Shanghai, China, Cat. No. D806297) for IBD model induction; chloral hydrate (Shanghai Maclin Biochemical Technology Co., Ltd., Shanghai, China, MFCD00044479) for mouse sedation; 4% paraformaldehyde (Beijing Solarbio Science & Technology Co., Ltd., Beijing, China, AR1069) for tissue fixation; IFX (provided by Jiangsu Second Hospital of Chinese Medicine, Nanjing, China) as the anti-TNF-α agent; phosphate-buffered saline (PBS; Nanjing Shengxing Bio-Tech Co., Ltd., Nanjing, China, SN331); a hematoxylin and eosin (H&E) staining kit (Shanghai Beyotime Biotechnology, Shanghai, China, C0105M); a mouse TNF-α ELISA kit (Wuhan Elabscience, Wuhan, China, E-EL-M3063); a mouse IL-6 ELISA kit (Wuhan Elabscience, Wuhan, China, E-EL-M0044c); total RNA extraction, reverse transcription, and qPCR detection kits (Novizan Biotech, Nanjing, China); DEPC-treated water (Beyotime Biotechnology, Shanghai, China); and all qPCR primers (GenScript Biotech, Nanjing, China). All reagents were stored and handled according to the manufacturers’ instructions.

### 2.2. Animal Experiments

#### 2.2.1. Establishment of the DSS-Induced Colitis Model

Ten-week-old male C57BL/6 mice (SPF grade; Yangzhou University Comparative Medicine Center, Yangzhou, China; license no. SCXK[Su]2023-0019) were acclimated for one week with ad libitum access to food and water. A subacute colitis model was induced by administering DSS (2% *w*/*v*) dissolved in sterile drinking water for 10 consecutive days [21]. This single-cycle protocol produces sustained colonic inflammation and systemic metabolic consequences that last several weeks, which makes this method suitable for evaluating exercise and biologic interventions. To validate successful model establishment, an additional 3 mice were subjected to the same DSS treatment prior to the formal experiment. These mice were euthanized on day 10, and colon tissues were collected for H&E staining. Histopathological examination confirmed mucosal ulceration, crypt architectural disruption, and inflammatory cell infiltration, verifying successful model induction. During modeling, body weight, stool consistency, and the presence of fecal blood were recorded daily. Successful model establishment was confirmed by persistent histopathological evidence of colonic inflammation—namely, mucosal ulceration, crypt architectural disruption, and inflammatory cell infiltration. The experimental protocols were approved by the Institutional Animal Care and Use Committee (IACUC) of Hubei Baithe Biotechnology Co., Ltd. (Wuhan, China; approval No. BNT-2024-023) and were carried out in accordance with the “Guide for the Care and Use of Laboratory Animals” (8th edition, NRC 2011), ensuring humane treatment at all times.

#### 2.2.2. Experimental Grouping and Interventions

Following successful model establishment, thirty mice were matched by body weight and disease activity index (DAI) and then randomly divided into five groups (*n* = 6 per group). This sample size was selected based on prior studies using similar DSS colitis and rodent resistance training models that detected meaningful differences with comparable or smaller group sizes [22,23].

**Figure 1:**
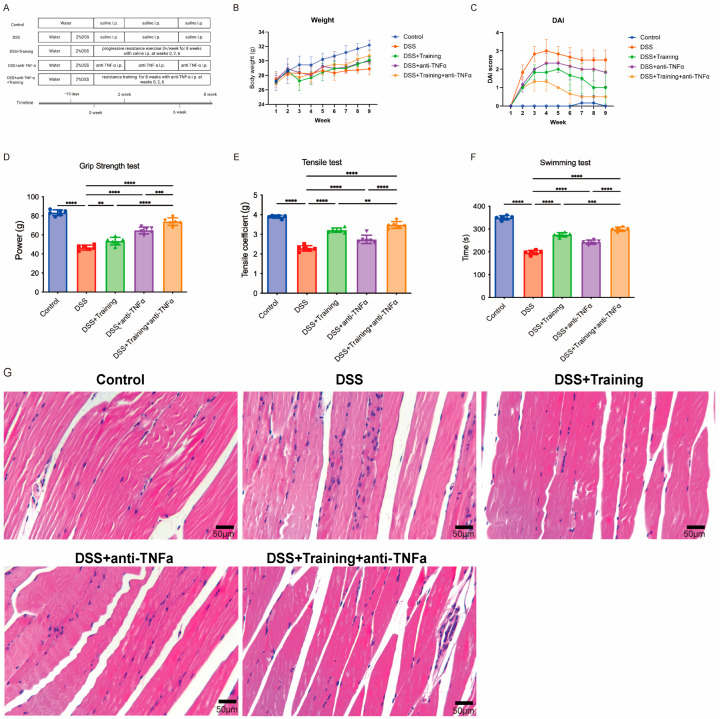
Resistance training combined with anti-TNF-α improves physiological function in mice with DSS-induced colitis. (**A**) Flow chart of the experiment. (**B**) Body weight trajectories over 8 weeks. (**C**) DAI scores. (**D**) Forelimb grip strength measurements. (**E**) Pull-strength coefficients. (**F**) Swimming endurance times. (**G**) Representative H&E-stained quadriceps muscle sections (scale bar = 50 μm). Data are presented as the mean ± SD ((**B**–**G**), *n* = 6). Statistical significance was determined by one-way ANOVA with Bonferroni post hoc test. ** *p* < 0.01, *** *p* < 0.001, **** *p* < 0.0001.

Randomization was performed by an investigator not involved in outcome assessments using a computer-generated random number sequence, and group assignments were concealed in sequentially numbered, opaque sealed envelopes until intervention commencement. The five groups were as follows: control group (CTRL), receiving sterile drinking water and intraperitoneal injections of sterile saline (0.9% NaCl) at weeks 0, 2, and 6 as the vehicle control for anti-TNF-α therapy; model control group (DSS), receiving intraperitoneal saline at weeks 0, 2, and 6 after DSS induction; resistance training group (DSS + training), undergoing progressive resistance training three times per week for 8 weeks post-DSS and receiving intraperitoneal saline at the same time points; anti-TNF-α treatment group (DSS + anti-TNF-α), receiving intraperitoneal injections of infliximab (5 mg/kg) at weeks 0, 2, and 6; and combination therapy group (DSS + anti-TNF-α + training), receiving both resistance training and infliximab on the same schedules (Figure 1A). The infliximab dose of 5 mg/kg and the administration schedule were selected based on previously reported therapeutic efficacy in murine colitis models, which have demonstrated that intravenous or intraperitoneal dosing at this level achieves adequate systemic exposure and neutralizes TNF-α activity for 2–4 weeks [24,25]. Although infliximab is a chimeric anti-human TNF-α antibody, it has been shown to exhibit biologically relevant cross-reactivity with murine TNF-α in these experimental systems, as evidenced by attenuation of proinflammatory cytokine production and histological damage.

**Figure A1:**
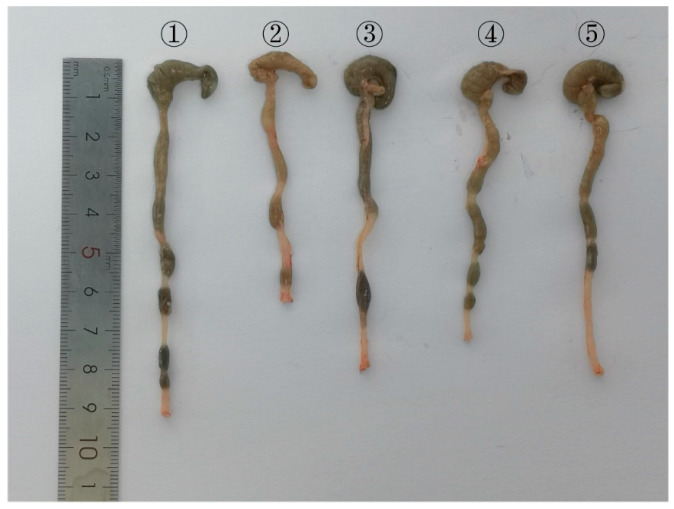
Representative gross morphology of the colon from each experimental group. (1) Control, (2) Model control (DSS), (3) Resistance training (DSS + Training), (4) Anti-TNF-α treatment (DSS + anti-TNF-α), and (5) Combination therapy (DSS + anti-TNF-α + Training). Images illustrate the macroscopic mucosal appearance across groups and were chosen to represent typical findings for each treatment arm.

At the end of the 8-week intervention, the mice were euthanized, and peripheral blood was collected for serum preparation. For anesthesia during tissue collection, mice were anesthetized with isoflurane inhalation (3–4% for induction, 1.5–2% for maintenance) using a calibrated vaporizer. At the endpoint, euthanasia was performed using CO_2_ inhalation followed by cervical dislocation to ensure death, consistent with AVMA-approved humane euthanasia methods. Colon mucosa and skeletal muscle tissues (including the quadriceps and gluteus maximus) were harvested and either fixed or snap-frozen for subsequent histological and molecular analyses. The representative gross morphology of the colon in each experimental group is shown in Figure A1.

The resistance training protocol was adapted from established ladder-climbing models in rodents [26], using a 1 m ladder inclined at approximately 80° with 2 cm rung spacing. All mice were acclimated to the facility and gently handled for one week prior to the experiments. The resistance training protocol began with a one-week familiarization phase of unloaded ladder climbing to minimize task-related stress, followed by eight weeks of progressive training. Tail weights were attached using foam-lined clamps, and loads were increased by 10% body weight weekly to avoid acute overexertion, in accordance with recommendations for minimizing non-specific stress in rodent exercise studies. We affixed tail weights (5–40 g; Lakeshore, Carson, CA, USA) via foam-lined nasal clamps (7.5 g; Harvard Apparatus, Holliston, MA, USA) and gently stimulated the tail to encourage climbing. Initial loading was set at 10% of body weight and increased by 10% every week until a maximum of 70% was reached, at which point the load increments ceased. Each training session comprised five climbs, performed three times per week (Monday, Wednesday, and Friday), following standardized timing and rest intervals [27]. These parameters—load progression, repetition count, and frequency—closely mirror validated rodent resistance training regimens known to induce muscle hypertrophy and mitochondrial adaptation.

#### 2.2.3. Body Weight, DAI, Muscle Strength, and Physical Performance Assessments

Body weight was recorded weekly for all mice using a precision electronic scale. The DAI was assessed daily during the DSS induction phase and included three clinical parameters: body weight loss, stool consistency, and fecal bleeding. Scoring was based on established criteria [28,29]: weight loss was scored as 0 (none), 1 (1–5%), 2 (5–10%), 3 (10–20%), or 4 (>20%); stool consistency was scored as 0 (normal formed pellets), 2 (loose stool), or 4 (diarrhea); and bleeding was scored as 0 (no blood), 1 (positive occult blood), 2 (positive occult blood with visible granular blood), or 4 (gross bleeding) (Table 1). The cumulative DAI for each mouse was calculated as the sum of the individual subscores divided by 3.

Forelimb grip strength was measured using a digital force gauge. The device was placed on a horizontal platform, and each mouse was suspended by the tail and allowed to grasp the sensor bar with its forepaws. The tail was then gently pulled backward until the mouse released the bar, at which point the peak force recorded by the gauge was taken as the maximum grip strength. Each mouse underwent three consecutive trials, and the highest value was recorded as the absolute forelimb grip strength [30,31]. For the hanging-rope pull test, a 1 m long rope 0.2 mm in diameter was suspended vertically, and each mouse was placed on the rope approximately 1 m above the floor. The latency to fall was recorded over three trials and averaged and then normalized to body weight to calculate the pull-strength coefficient [32]. Swimming endurance was assessed in a circular tank (diameter, 0.5 m; water depth, ≥30 cm; temperature, ~25 °C). After a 3 min adaptation swim, the mice were fitted with a lead weight equivalent to 5% of their body weight at the proximal third of the tail and allowed to swim. The time until the animal could no longer maintain buoyancy—defined by erratic movements and failure to resurface within 7 s—was recorded up to a maximum of 5 min and taken as the swimming endurance time [33]. All functional assays were conducted at the same time of day during the final three days of the intervention period, and all assessments were performed by an investigator blinded to group allocation to minimize bias.

Upon euthanasia, the quadriceps muscles were rapidly dissected from both hindlimbs, blotted free of blood and visible connective tissue, and weighed on a precision analytical balance to the nearest milligram. Muscle mass was recorded as absolute wet weight (mg).

#### 2.2.4. Histology

Quadriceps and colon samples were harvested from all mice in each group (*n* = 6 per group). Upon euthanasia, the entire colon was excised from the cecocolonic junction to the anal verge. Colon length was measured to the nearest millimeter using a standard ruler by an investigator blinded to group allocation. Colon shortening was used as a macroscopic indicator of colitis severity.

The selected tissues were fixed in 4% paraformaldehyde (Boster Biological Technology, Beijing, China, AR1069) for 24 h, then processed in a Leica ASP300 tissue processor through graded ethanol dehydration (75%, 85%, 90%, and 95% each for 1 h), two changes in absolute ethanol (1 h each), two changes in xylene (30 min each), and three paraffin infiltrations (1 h each), after which the samples were embedded in molten paraffin, precooled to −20 °C, and allowed to harden at 4 °C. Paraffin blocks were cut into 4 μm sections on a Leica RM2235 microtome, floated on a 45 °C water bath, and mounted on glass slides. The sections were subsequently dried and fixed at 37 °C using a Leica HI1220 slide dryer. Sections were deparaffinized in xylene I–IV (5 min each), rehydrated through a descending ethanol gradient (100%, 90%, 80%, and 70% each for 5 min), and rinsed in running water. The sections were stained with hematoxylin for 10 min, differentiated in 1% acid alcohol for 5–10 s, rinsed in water for 10 min, and counterstained with eosin for 5 min. Finally, the sections were dehydrated in three changes in absolute ethanol (5 min each), cleared in xylene (5 min, twice), and coverslipped with neutral gum. All histological assessments were performed in a blinded manner to minimize potential bias. Specifically, all sections were randomly coded by an investigator who was not involved in the group allocation or experimental interventions, thereby concealing group identity. Subsequently, at least two trained investigators, blinded to the experimental groups, independently evaluated and scored the sections according to predefined criteria (Table A1). Muscle fiber architecture and colonic mucosal integrity were then examined and imaged under an Olympus BX51 light microscope, with representative images archived for subsequent morphometric analysis.

### 2.3. Serum Biochemistry

Frozen serum samples stored at –80 °C were thawed slowly at 4 °C for 12 h. After thawing, the samples were gently mixed by inversion and centrifuged at 3000 rpm for 10 min at 4 °C to remove any insoluble material. The resulting supernatant was assayed for lactate dehydrogenase (LDH) and creatine kinase (CK) using a fully automated biochemical analyzer (BK-280, BOKO Bio, Jinan, China). All reagents, calibrators, and quality control materials were used according to the manufacturer’s predefined protocols. Two-level quality control samples were measured at the beginning of each analytical run, and sample measurements were accepted only when both QC levels fell within the specified ranges. All samples were measured in a single batch to avoid inter-batch variation, and the operator was blinded to group allocation. The instrument software directly calculated analyte concentrations.

### 2.4. ELISA

Frozen serum samples stored at −80 °C were thawed at 4 °C for 12 h, equilibrated to room temperature for 20 min, and then centrifuged at 3000 r/min for 10 min at 4 °C to remove particulates. Mouse TNF-α and IL-6 levels were quantified using paired ELISA kits (E-EL-M3063 and E-EL-M0044c; Elabscience, Wuhan, China) with all reagents prewarmed to room temperature for 20 min. Standards (S0–S5, with S0 as blank) were prepared by serial dilution according to the kit instructions, and 1× wash buffer was prepared from 20× stock using deionized water. In the precoated microplate, 50 μL of standard was added to standard wells, while sample wells received 10 μL of appropriately diluted serum followed immediately by 40 μL of sample diluent to achieve a 1:5 dilution; blank wells received no sample. After 100 μL of HRP-conjugated detection antibody was added to each well (excluding blank wells), the plates were sealed and incubated at 37 °C for 60 min. The wells were then washed five times with 1× wash buffer (1 min soak per wash) and blotted dry. Subsequently, 50 μL each of substrate solutions A and B was added, and the plate was incubated in the dark at 37 °C for 15 min, after which 50 μL of stop solution was added. Optical density (OD) was measured within 15 min at 450 nm on a Multiskan™ GO microplate reader (Thermo Fisher Scientific, Waltham, MA, USA). A standard curve was generated by plotting known standard concentrations against OD values, and sample concentrations were interpolated using the resulting linear regression equation. All samples were assayed in duplicate to ensure accuracy.

### 2.5. Muscle Transcriptome Sequencing and qPCR Validation

Quadriceps muscles from each experimental group were flash-frozen in liquid nitrogen and ground to a fine powder, after which total RNA was extracted using a commercial RNA isolation kit. For qPCR validation, to enhance statistical power, samples from four mice per group were used, comprising the three sequencing cohort mice plus one additional mouse randomly selected from the remaining molecular samples (*n* = 4 per group). RNA concentration and integrity were assessed by NanoDrop spectrophotometry and an Agilent 2100 Bioanalyzer (RIN > 7.5 for all samples). Libraries were prepared using poly(A) enrichment, fragmented, reverse-transcribed, and adapter-ligated; all libraries were constructed and sequenced in a single batch to eliminate batch effects. Paired-end sequencing (150 bp) was performed on an Illumina NovaSeq 6000 platform. Raw reads were filtered for quality and mapped to the mouse reference genome (GRCm38) using HISAT2. Gene expression counts were normalized using the trimmed mean of M-values (TMM) method (edgeR) and expressed as fragments per kilobase of transcript per million mapped reads (FPKM) and transcripts per million (TPM). Differentially expressed genes (DEGs) were identified using the limma-voom pipeline with empirical Bayes moderation; genes with a |fold change| > 1.5 and a Benjamini–Hochberg-adjusted false discovery rate (FDR) < 0.05 were considered significant. DEGs were subjected to hierarchical clustering, Gene Ontology (GO) enrichment, and Kyoto Encyclopedia of Genes and Genomes (KEGG) pathway analysis using clusterProfiler, with *p*-values corrected by the Benjamini–Hochberg method.

For qPCR validation, the same RNA samples were treated with gDNA remover and reverse-transcribed to cDNA using HiScript III SuperMix. Quantitative real-time PCR was performed on an ABI StepOnePlus system with ChamQ Universal SYBR qPCR Master Mix, and β-actin was used as the internal control. The cycling conditions were 95 °C for 30 s, followed by 40 cycles of 95 °C for 10 s and 60 °C for 30 s, with a subsequent melt–curve analysis. Relative expression levels were calculated using the 2^−ΔΔCt^ method. Table A2 lists the primer sequences that were used.

### 2.6. Statistical Analysis

Data are presented as mean ± standard error of the mean (SEM) for normally distributed continuous variables, and as median with interquartile range (IQR) for non-normally distributed or ordinal variables. Statistical analyses were performed using GraphPad Prism 9 and SPSS (version 27.0). The primary endpoints were forelimb grip strength, serum TNF-α, and serum IL-6. Secondary endpoints included all other functional, biochemical, histological, and molecular outcomes.

Normally distributed continuous data were compared among the five groups by one-way ANOVA with Bonferroni post hoc test, after confirming normality and homogeneity of variances. Non-normally distributed or ordinal data were analyzed using the Kruskal–Wallis test followed by Dunn’s post hoc test with Bonferroni correction.

To evaluate the main effects of resistance training, anti-TNF-α treatment, and their interaction, a two-way ANOVA was conducted using a 2 × 2 factorial design restricted to the four DSS-induced groups (DSS, DSS + training, DSS + anti-TNF-α, DSS + training + anti-TNF-α), with training and anti-TNF-α entered as fixed factors. When a significant interaction was detected, simple effects analyses were performed, and multiple comparisons were adjusted using Šídák’s post hoc correction.

Longitudinal data (body weight and DAI) were analyzed using two-way repeated-measures ANOVA with group as a between-subjects factor and time as a within-subjects factor. Sphericity was assessed using Mauchly’s test; where violated, the Greenhouse–Geisser correction was applied, and corrected degrees of freedom are reported.

All statistical tests were two-sided, and *p* < 0.05 was considered statistically significant.

## 3. Results

### 3.1. DSS-Induced Colitis Is Associated with Reduced Muscle Strength and Function, and Combined Resistance Training and Anti-TNF-α Therapy Improves Systemic Phenotypes and Muscle Performance in Colitis Model Mice

#### 3.1.1. DSS-Induced Sarcopenia-like Phenotype

**Figure 2:**
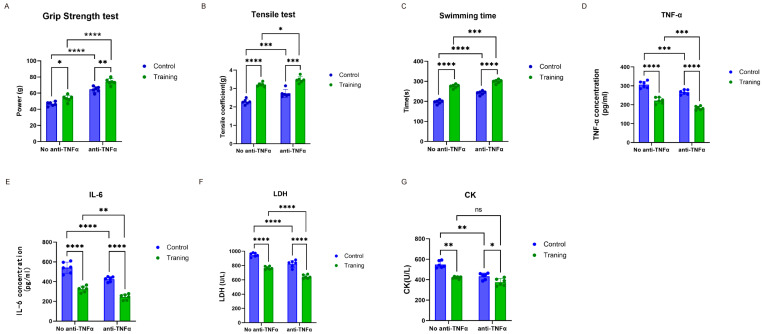
Two-way ANOVA of the effects of resistance training and anti-TNF-α therapy on muscle function and serum markers in IBD model mice. (**A**) Forelimb grip strength; (**B**) tensile test; (**C**) swimming time; (**D**) serum TNF-α; (**E**) serum IL-6; (**F**) serum LDH; (**G**) serum CK. The data are presented as the mean ± SD (*n* = 6). * *p* < 0.05, ** *p* < 0.01, *** *p* < 0.001, **** *p* < 0.0001.

Over the 8-week experimental period, body weight, DAI, grip strength, pulling force coefficient, and swimming endurance were dynamically monitored across all groups (Figure 1B–F and Figure 2A–C, Table A3). Changes in body weight over time were analyzed using a two-way repeated-measures ANOVA. Mauchly’s test indicated a violation of sphericity (W = 0.001, *p* < 0.001); therefore, the Greenhouse–Geisser correction (ε = 0.510) was applied. The analysis revealed a highly significant main effect of time (F(4.076, 101.912) = 44.889, *p* < 0.0001), as well as a significant main effect of treatment (F(4, 25) = 16.928, *p* < 0.0001), indicating an overall temporal change in body weight and substantial differences among treatment groups. However, the interaction between time and treatment did not reach statistical significance (F(16.306, 101.912) = 2.409, *p* = 0.076), suggesting largely parallel trajectories of body weight change among groups over time. During the mid-to-late stages of model induction, mice in the DSS group exhibited persistently lower body weight compared with healthy controls. By week 8, the mean difference was 3.285 g (95% CI [1.725, 4.845], *p* < 0.001), confirming progressive disease-associated wasting. A two-way repeated-measures ANOVA of DAI scores was also conducted. Mauchly’s test indicated a violation of sphericity (W = 0.008, *p* < 0.001), and the Greenhouse–Geisser correction (ε = 0.417) was applied. The analysis demonstrated a significant interaction between group and time (F(13.330, 83.315) = 7.222, *p* < 0.0001), indicating a time-dependent and group-specific dynamic pattern of disease activity. The main effect of group was robust (F(4, 25) = 46.34, *p* < 0.0001), underscoring treatment group as a dominant determinant of disease progression. Notably, DSS-treated mice exhibited significantly elevated DAI scores compared with healthy controls from the first week onward, confirming sustained colonic inflammation throughout the experimental period.

In addition, compared with healthy controls, DSS-induced model mice exhibited a marked reduction in grip strength (*p* < 0.0001) and pulling force coefficient (*p* < 0.0001), accompanied by a significant shortening of swimming endurance time (*p* < 0.0001). These findings indicate that DSS treatment successfully induced systemic wasting and exercise intolerance characteristic of IBD. Notably, we observed an apparent dissociation between sustained elevation of DAI and the relatively modest body weight changes in DSS-treated mice during the post-DSS recovery phase. The sustained elevation of the DAI despite modest body weight changes reflects the composite nature of the DAI score, wherein compensatory hyperphagia and restored absorptive function following DSS withdrawal facilitate weight recovery, while persistent mucosal injury sustains elevated stool consistency and fecal bleeding subscores—a dissociation between systemic metabolic adaptation and local colonic inflammation that is well documented in single-cycle DSS models [34,35]. Consistent with these functional impairments, histological analysis (Figure 1G) revealed tightly organized muscle fibers with preserved cellular architecture in control mice, whereas DSS-treated mice displayed prominent inflammatory cell infiltration and muscle fiber disruption or degeneration, supporting the presence of a sarcopenia-like pathological phenotype. Quantitative assessment confirmed the structural deficits in DSS-treated mice. Quadriceps muscle weight differed significantly among groups (one-way ANOVA: F(4, 25) = 57.94, *p* < 0.001). The DSS group had significantly lower muscle weight compared with the control group (*p* < 0.001), confirming substantial muscle wasting (Table A4). Collectively, these data demonstrate that DSS-induced colitis is associated with reduced muscle mass and functional impairment.

#### 3.1.2. Combined Resistance Training and Anti-TNF-α Therapy Improves Physiological Function and Muscle Morphology in Mice with DSS-Induced Colitis

Building on the DSS-induced colitis model, both resistance training and anti-TNF-α therapy partially mitigated systemic wasting and functional decline, whereas their combination resulted in superior benefits. Specifically, compared with DSS alone, DSS + training, DSS + anti-TNF-α, and DSS + training + anti-TNF-α tended to support body weight recovery, with the greatest increase observed in the combined therapy group. The DAI decreased significantly in the resistance training and training + anti-TNF-α groups, whereas the anti-TNF-α group showed a downward trend that did not reach statistical significance.

Muscle function assessments further delineated the independent and combined effects of the interventions (Table A5). Two-way ANOVA revealed highly significant main effects of both anti-TNF-α treatment (*p* < 0.0001) and resistance training (*p* = 0.0012) on grip strength, indicating that each intervention independently improved muscle strength. No significant interaction between the two factors was detected. Šídák post hoc analyses showed that, regardless of anti-TNF-α treatment status, mice subjected to resistance training exhibited significantly greater grip strength than their non-trained counterparts under the same conditions (all *p* < 0.01).

Two-way ANOVA of pulling force confirmed highly significant main effects of both anti-TNF-α treatment (*p* < 0.0001) and resistance training (*p* < 0.0001), indicating that each intervention independently and decisively improved muscle endurance. The interaction between the two factors was not significant. Šídák-adjusted post hoc tests further established the robustness of the training effect: regardless of anti-TNF-α treatment status, trained mice showed markedly greater pulling force than their non-trained counterparts under the same inflammatory condition (all *p* < 0.001). Specifically, resistance training increased the mean pulling-force coefficient by 0.8350 units (95% CI: 0.6613 to 1.009, *p* < 0.0001), while anti-TNF-α treatment produced an increase of 0.3583 units (95% CI: 0.2416 to 0.4751, *p* < 0.0001).

For swimming endurance, two-way ANOVA showed that both resistance training and anti-TNF-α treatment had highly significant main effects (both *p* < 0.0001), and a statistically significant interaction between the two factors was detected. Šídák post hoc comparisons indicated that resistance training produced a highly significant increase in swim time under both inflammatory conditions (both *p* < 0.0001). Simple-effects analysis demonstrated that resistance training markedly improved exercise endurance in DSS-treated mice and that the magnitude of this improvement was larger under higher inflammatory load; the between-condition difference in training-induced improvement was approximately 78.0 s (95% CI: 66.9–89.1 s, *p* < 0.001), indicating that training confers greater gains in endurance when systemic inflammation is more severe.

Histological analysis (H&E staining) corroborated these functional findings. In the resistance training group, the morphology of the muscle fibers was nearly normal, and inflammatory infiltrates were almost completely resolved. The anti-TNF-α group displayed moderate nuclear proliferation with residual, mild inflammation. Remarkably, the training + anti-TNF-α group exhibited muscle fibers indistinguishable from those in the control group, with a well-preserved architecture and no detectable inflammatory cells. Both resistance training and anti-TNF-α therapy partially attenuated DSS-induced muscle loss. The DSS + Training group (*p* < 0.001) and the DSS + anti-TNF-α group (*p* = 0.004) showed significantly greater muscle weight than the DSS group. The combined intervention produced the greatest preservation of muscle mass (*p* < 0.001), which did not differ significantly from the control group (*p* = 0.106), indicating near-complete preservation of muscle mass (Table A4). Taken together, these results indicate that resistance training and anti-TNF-α therapy each contribute independently to ameliorating systemic and muscular deficits in this model. Their combination, through additive effects on multiple endpoints, produced the most pronounced overall improvement.

### 3.2. DSS-Induced Colitis Is Associated with Exacerbated Systemic Inflammation and Widespread Tissue Injury, Which Are Attenuated by Resistance Training and Anti-TNF-α

#### 3.2.1. DSS-Induced Systemic Inflammation and Tissue Damage

**Figure 3:**
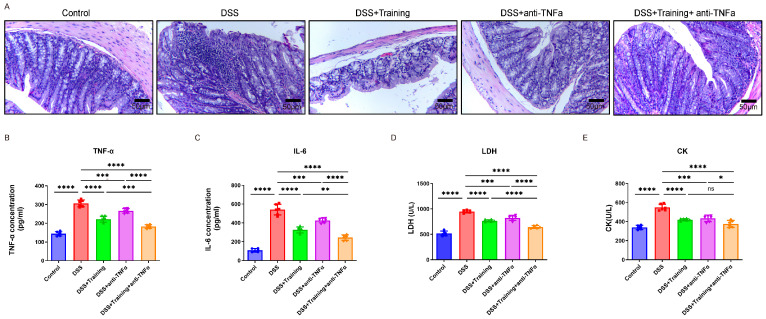
Combined resistance training and anti-TNF-α attenuate tissue damage and inflammation in mice with DSS-induced colitis. (**A**) H&E staining of colonic tissue from each experimental group. (**B**–**E**) Serum levels of the proinflammatory cytokines TNF-α and IL-6, along with the tissue injury markers LDH and CK, across the different treatment groups. The data are presented as the mean ± SD (*n* = 6). ns: not significant, * *p* < 0.05, ** *p* < 0.01, *** *p* < 0.001, **** *p* < 0.0001.

To determine whether DSS-induced colonic inflammation is accompanied by systemic inflammatory responses and tissue injury, we first performed H&E staining on colon sections from each experimental group (Figure 3A). In control mice, the colonic epithelium was intact, the crypt architecture remained orderly, and inflammatory cell infiltration was absent. In contrast, DSS-treated mice exhibited severe mucosal damage, with multiple erosions, superficial ulcerations, disrupted crypt organization, and dense inflammatory infiltrates, indicating a robust local inflammatory response. Macroscopic assessment confirmed the presence of severe colonic inflammation in DSS-treated mice. Colon length differed significantly among groups (F(4, 25) = 42.17, *p* < 0.001). The DSS group exhibited significantly shorter colon length compared with the control group (*p* < 0.001), consistent with marked colonic inflammation and tissue remodeling (Table A4).

We next quantified systemic inflammation and tissue damage by measuring serum levels of proinflammatory cytokines (TNF-α and IL-6) and injury markers (LDH and CK) (Figure 3B–E). Compared with control mice, model mice exhibited profound increases in serum IL-6 (*p* < 0.0001) and TNF-α levels (*p* < 0.0001), reflecting an exacerbated systemic proinflammatory state. Concurrently, serum LDH (*p* < 0.0001) and CK (*p* < 0.0001) levels were significantly increased, indicating widespread tissue injury and disrupted muscle metabolism (Table A6).

#### 3.2.2. Combined Resistance Training and Anti-TNF-α Therapy Significantly Attenuates Inflammatory Responses and Tissue Damage in DSS-Induced Mice

Building on the identification of pronounced local and systemic pathology in DSS-treated mice, we next evaluated the therapeutic effects of resistance training, anti-TNF-α, and their combination. Histological analysis demonstrated that compared with DSS alone, all three interventions resulted in marked amelioration of colonic damage: the mucosal architecture was progressively restored, crypt organization was normalized, and inflammatory infiltrates were substantially reduced, indicating partial repair of DSS-induced epithelial injury. Blinded semi-quantitative histological scoring confirmed these observations. The total histological score (sum of inflammatory cell infiltration, crypt architecture disruption, and mucosal ulceration/erosion; range 0–12) differed significantly among the five groups (Kruskal–Wallis χ^2^ = 26.839, *p* < 0.001). The DSS group showed significantly higher scores than the control group (*p* < 0.001). Dunn’s post hoc test with Bonferroni correction revealed that total histological scores were significantly reduced in the DSS + Training + anti-TNF-α group compared with the DSS group (*p* = 0.004). The DSS + anti-TNF-α group also trended lower than the DSS group, though this difference did not reach statistical significance after correction (*p* = 0.446). The DSS + Training group did not differ significantly from DSS (*p* = 1.000). Notably, the combination group did not differ significantly from the control group (*p* = 1.000), indicating near-complete histological normalization (Table A7). All three interventions partially attenuated DSS-induced colon shortening. Colon length was significantly greater in the DSS + Training group (*p* = 0.001), the DSS + anti-TNF-α group (*p* < 0.001), and the DSS + Training + anti-TNF-α group (*p* < 0.001). The combination group showed values closest to those of healthy controls, although the difference between the combination group and controls remained statistically significant (*p* = 0.035), suggesting substantial but incomplete restoration of colon length (Table A4).

Concomitantly, both resistance training and anti-TNF-α monotherapies significantly decreased serum TNF-α (resistance training: *p* < 0.0001; anti-TNF-α: *p* < 0.001) and IL-6 levels (all *p* < 0.0001), reflecting effective dampening of systemic inflammation. Markers of tissue injury—serum LDH (all *p* < 0.0001) and CK (all *p* < 0.0001)—were also decreased significantly in each treatment group compared with those in the DSS alone group, indicating improved cellular integrity and muscle metabolic status. Notably, the training + anti-TNF-α combination achieved the greatest reduction across all measures (all *p* < 0.0001).

Across a panel of serum biomarkers, two-way ANOVA revealed a consistent pattern (Figure 2D–G, Table A5): resistance training exerted an independent and dominant effect in attenuating systemic inflammation and tissue injury. For the proinflammatory cytokines TNF-α and IL-6, the training factor was the primary determinant of circulating levels (both *p* < 0.001). Likewise, resistance training produced highly significant main effects on the tissue-injury markers LDH and CK (both *p* < 0.05). In all analyses, the interaction between training and anti-TNF-α treatment was non-significant (all *p* > 0.05), indicating that the two interventions reduced circulating cytokines and injury markers in an independent, additive manner. Šídák-adjusted post hoc tests confirmed the robustness of the training effect: irrespective of anti-TNF-α treatment status, resistance training markedly lowered serum TNF-α, IL-6, and LDH (all *p* < 0.01). For the muscle-specific marker CK, training produced a highly significant reduction in mice that did not receive anti-TNF-α (*p* = 0.0019); in mice receiving anti-TNF-α, there was a downward trend in CK with training that did not reach statistical significance, suggesting that the training-associated benefit on muscle injury may be most pronounced under higher inflammatory load.

Integrating histological and circulating marker data, these findings demonstrate that DSS-induced colitis is accompanied by marked systemic inflammation and tissue damage and that resistance training is a potent, systemic anti-inflammatory and tissue-protective intervention. The training effect was largely independent of anti-TNF-α therapy, providing experimental evidence that exercise can reduce inflammatory burden and tissue injury in a manner that is additive to biologic treatment.

### 3.3. Transcriptomic Profiling Reveals Suppression of Oxidative Phosphorylation Genes by DSS and Suggests That Combined Resistance Training and Anti-TNF-α Therapy May Restore Mitochondrial Oxidative Metabolism While Suppressing Inflammatory Signaling

#### 3.3.1. DSS-Induced Downregulation of Oxidative Phosphorylation Pathway Genes in Skeletal Muscle

**Figure 4:**
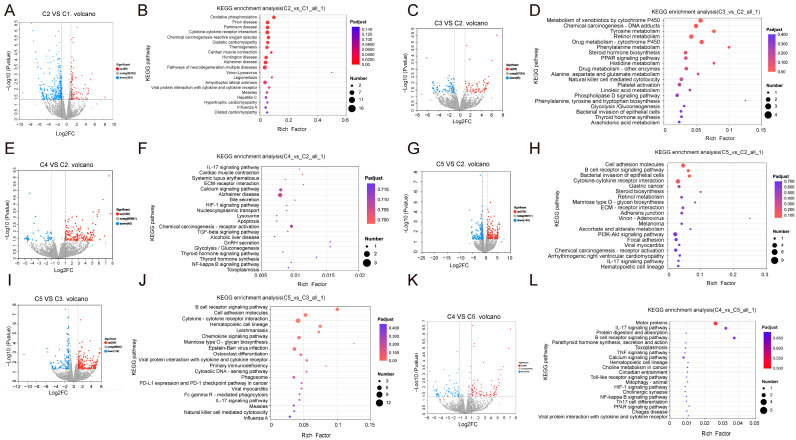
Combined resistance training and anti-TNF-α therapy enhances therapeutic efficacy by restoring oxidative phosphorylation gene expression and inhibiting inflammatory pathways. (**A**,**B**) Volcano plot and KEGG pathway enrichment of DEGs in C2 versus C1. (**C**,**D**) Volcano plot and KEGG enrichment of DEGs in C3 versus C2. (**E**,**F**) Volcano plot and KEGG enrichment of DEGs in C4 versus C2. (**G**,**H**) Volcano plot and KEGG enrichment of DEGs in C5 versus C2. (**I**,**J**) Volcano plot and KEGG enrichment of DEGs in C5 versus C3. (**K**,**L**) Volcano plot and KEGG enrichment of DEGs in C5 versus C4. C1: control group; C2: DSS model group; C3: resistance training group; C4: anti-TNF-α-treated group; C5: combined intervention group.

To elucidate the molecular sequelae of DSS-induced colitis and subsequent interventions, we performed high-throughput RNA sequencing on quadriceps muscle from each experimental group. Differential expression analysis, employing stringent criteria (|fold change| > 1.5 and FDR < 0.05), revealed a cohort of genes with significantly altered expression in the DSS model group (C2) versus the control group (C1). Among these genes, the most pronounced transcriptional suppression was observed for genes encoding components of the mitochondrial oxidative phosphorylation (OXPHOS) machinery (Figure 4A). KEGG pathway enrichment further confirmed that C2 exhibited marked downregulation of pathways essential for ATP synthesis, proton-pump activity, and redox balance (Figure 4B). These data provide a molecular basis for inferring that DSS-induced colitis is associated with transcriptional suppression of mitochondrial energy metabolism in skeletal muscle, underscoring the systemic metabolic derangements associated with IBD at the gene expression level and establishing a critical foundation for assessing the efficacy of targeted interventions aimed at restoring mitochondrial gene programs.

#### 3.3.2. Combined Resistance Training and Anti-TNF-α Therapy Restores Mitochondrial Oxidative Phosphorylation Gene Expression, Reprograms Metabolic Pathways, and Suppresses Multiple Proinflammatory Signals

Comparison between the resistance training group (C3) and the DSS model group (C2) revealed that resistance training markedly altered the transcriptional profile of metabolic genes in skeletal muscle. KEGG enrichment analysis of DEGs (FDR < 0.05) demonstrated significant enrichment in the PPAR signaling pathway, as well as tyrosine, retinol, and phenylalanine metabolism pathways (Figure 4C,D). These results indicate broad transcriptional changes affecting energy metabolism-related genes and upstream fatty acid and amino acid metabolic pathways.

Differential expression analysis between the anti-TNF-α-treated group (C4) and DSS model group (C2) showed that genes in C4 were significantly enriched in pathways related to inflammation and tissue remodeling, including the IL-17 signaling pathway, extracellular matrix (ECM)–receptor interactions, HIF-1 signaling, and TGF-β signaling (Figure 4E,F). These findings demonstrate that anti-TNF-α treatment is associated with transcriptional regulation of gene sets involved in inflammatory response modulation and tissue repair processes.

In the combined intervention group (C5), comparison with the DSS model group (C2) revealed that DEGs were significantly enriched in multiple metabolism- and inflammation-related pathways, including cell adhesion molecules (CAMs), retinol metabolism, steroid biosynthesis, IL-17 signaling, PI3K-Akt signaling, and cytokine–cytokine receptor interactions (Figure 4G,H). Compared with the resistance training-only group (C3), DEGs in C5 showed distinct enrichment patterns within immune- and inflammation-related pathways, including B-cell receptor signaling, chemokine signaling, and PI3K-Akt signaling (Figure 4I,J), indicating a transcriptional response unique to the combined intervention.

Differential expression analysis between the combined intervention group (C5) and the anti-TNF-α therapy-only group (C4) identified approximately 167 significant DEGs. KEGG enrichment analysis demonstrated that these genes were significantly associated with canonical inflammation- and immunity-related pathways, including IL-17 signaling, TNF signaling, Toll-like receptor signaling, and NF-κB signaling (FDR < 0.01; Figure 4K,L).

Overall, relative to the healthy control group, the DSS model group exhibited a general downregulation of oxidative phosphorylation-related gene sets and upregulation of inflammation-related gene sets. Resistance training alone led to upregulation of a subset of metabolism-related genes, whereas anti-TNF-α treatment was associated with downregulation of inflammation-related genes. The combined intervention displayed transcriptional features distinct from either single intervention, suggesting that resistance training and anti-TNF-α treatment collectively modulate multiple gene sets to influence the molecular phenotype of muscle tissue.

### 3.4. qPCR Validation Confirms That Combined Resistance Training and Anti-TNF-α Restores Mitochondrial Oxidative Phosphorylation Gene Expression and Suppresses Proinflammatory Gene Expression in DSS-Induced Mice

#### 3.4.1. DSS-Induced Mitochondrial Energy Metabolism Impairment and Proinflammatory Gene Activation

To validate the transcriptomic and KEGG enrichment findings, we quantified the expression of mitochondrial oxidative phosphorylation genes (ND1, ND2, ND3, ND4, ND4L, ND5, ND6, COX1, COX2, and Cyt b) and key inflammatory mediators (IFN-γ, IL-1β, IL-6, TNF-α, MMP9, S100a8, and S100a9) by qPCR. Compared with C1, C2 significantly downregulated the expression of all the mitochondrial genes (*p* < 0.01), confirming DSS-induced disruption of oxidative phosphorylation capacity. Concurrently, C2 robustly upregulated IFN-γ, IL-1β, IL-6, TNF-α, MMP9, and S100a8 expression (*p* < 0.001), whereas S100a9 expression remained unchanged. These results genetically substantiate the coexistence of mitochondrial dysfunction and systemic inflammatory amplification in muscle tissue of mice with DSS-induced colitis.

#### 3.4.2. Combined Resistance Training and Anti-TNF-α Therapy Restore Oxidative Phosphorylation Gene Expression and Suppress Proinflammatory Gene Expression

**Figure 5:**
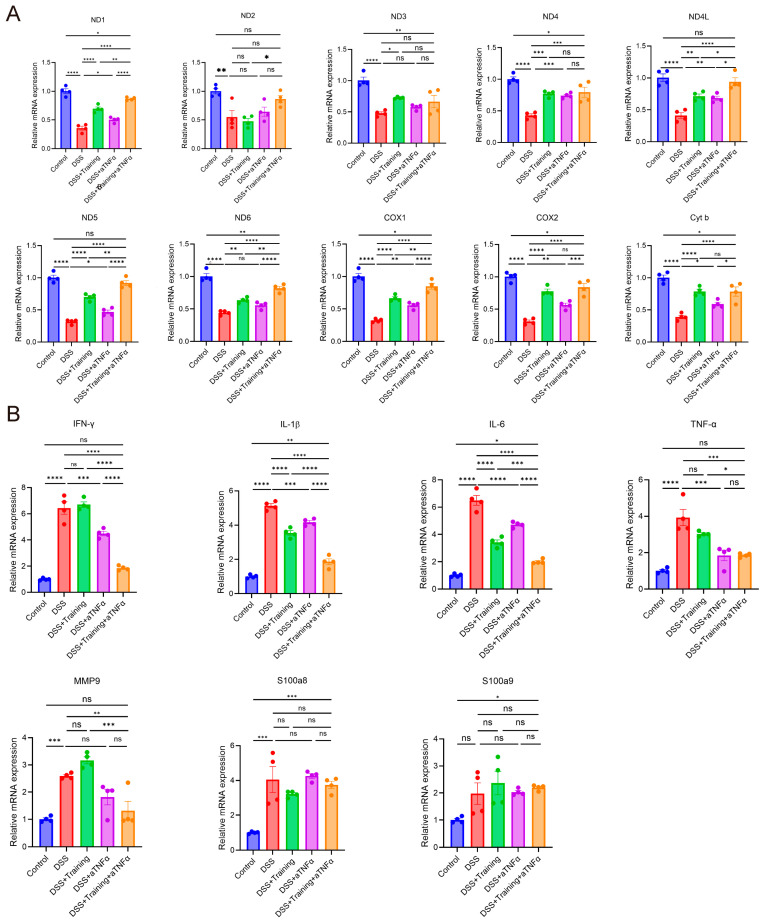
Combined resistance training and anti-TNF-α therapy enhances oxidative phosphorylation gene expression and significantly inhibits inflammatory pathways. (**A**) Relative mRNA expression of mitochondrial oxidative phosphorylation genes (ND1, ND2, ND3, ND4, ND4L, ND5, ND6, COX1, COX2, and Cyt b) in quadriceps muscle from each group. (**B**) Relative mRNA expression of proinflammatory cytokine genes (IFN-γ, IL-1β, IL-6, TNF-α, MMP9, S100a8, and S100a9) in each group. The data are presented as the mean ± SD (*n* = 4). ns: not significant, * *p* < 0.05, ** *p* < 0.01, *** *p* < 0.001, **** *p* < 0.0001.

To further validate the pathway enrichment results observed under resistance training and anti-TNF-α interventions, we assessed the mRNA expression levels of mitochondrial oxidative phosphorylation-related genes and proinflammatory genes in skeletal muscle across the intervention groups. Compared with C2, C3 showed significant upregulation of ND1, ND3, ND4, ND4L, ND5, ND6, COX1, COX2, and Cyt b, with increases in ND1, ND4, ND5, COX1, COX2, and Cyt b being particularly prominent (*p* < 0.001), indicating that resistance training partially restores mitochondrial energy metabolism. In C4, ND1, ND4, ND4L, ND5, COX1, COX2, and Cyt b, expression levels were also elevated relative to those in C2, with ND4 showing the most pronounced increase (*p* < 0.001), suggesting that anti-TNF therapy alone contributes to the recovery of oxidative phosphorylation gene expression. In C5, ND1, ND4, ND4L, ND5, ND6, COX1, COX2, and Cyt b levels were significantly upregulated compared with those in C2 (*p* < 0.001), and the expression levels of ND1, ND3, ND4, ND6, COX1, COX2, and Cyt b were only slightly reduced compared with those in C1 (*p* < 0.05), indicating that the combination therapy most effectively restores mitochondrial gene expression. Further comparisons revealed that the expression levels of ND1, ND2, ND4L, ND5, ND6, and COX1 were further upregulated in C5 compared with C3, and compared with those in C4, the expression levels of ND1, ND4L, ND5, ND6, COX1, COX2, and Cyt b were significantly greater in C5, with ND1, ND5, ND6, COX1, and COX2 exhibiting the most notable increases (*p* < 0.001) (Figure 5A). Taken together, these results indicate that resistance training effectively enhances mitochondrial gene expression, and anti-TNF therapy alone has a partial effect, whereas their combination clearly promotes mitochondrial gene recovery.

In terms of proinflammatory gene expression, compared with C2, C3 significantly downregulated IL-1β and IL-6 expression (*p* < 0.0001), indicating that resistance training partially suppresses DSS-induced inflammatory responses. The expression of IFN-γ, IL-1β, IL-6, and TNF-α was significantly lower in C4 than in C2 (*p* < 0.001), confirming the effectiveness of anti-TNF therapy in suppressing the secretion of inflammatory cytokines. The expression levels of IFN-γ, IL-1β, IL-6, TNF-α, and MMP9 were markedly lower in C5 than in C2 (*p* < 0.01), with those of IFN-γ, IL-1β, IL-6, and TNF-α decreasing the most (*p* < 0.001). However, compared with those in C1, the levels of IL-1β (*p* < 0.01) and IL-6 (*p* < 0.05) remained slightly elevated, and the level of S100a8 was significantly upregulated (*p* < 0.001), suggesting that while the combined intervention substantially inhibited inflammation, the baseline inflammation did not fully return to physiological levels. Further comparisons revealed that expression levels of IFN-γ, IL-1β, IL-6, TNF-α, and MMP9 were lower in C5 than in C3 (*p* < 0.05), with the reductions in IFN-γ, IL-1β, IL-6, and MMP9 being particularly pronounced (*p* < 0.001). Compared with C4, C5 demonstrated even stronger inhibition of IFN-γ, IL-1β, IL-6, and MMP9 (*p* < 0.0001), indicating a suppressive effect of resistance training combined with anti-TNF therapy on multiple proinflammatory signaling pathways (Figure 5B). Taken together, these results indicate that both resistance training and anti-TNF monotherapy effectively reduced the expression of several proinflammatory genes; however, their combination led to significantly enhanced suppression of key inflammatory mediators, such as IFN-γ, IL-1β, IL-6, TNF-α, and MMP9.

## 4. Discussion

IBD is a systemic disorder characterized by chronic intestinal inflammation, often accompanied by skeletal muscle wasting and functional impairment, which can severely affect patient outcomes. Using a DSS-induced colitis mouse model, this study systematically evaluated the effects of resistance training on skeletal muscle energy metabolism and systemic inflammatory status, as well as its combined effects with anti-TNF-α antibody treatment. The single-cycle DSS model used here, while not replicating advanced fibrotic disease, effectively induced a prolonged state of systemic immune-metabolic disturbance following acute colitis, consistent with established protocols [36,37]. Our data demonstrate sustained inflammation, tissue pathology, and muscle transcriptomic alterations eight weeks post-DSS, confirming a persistent pathological state. This model is thus suitable for evaluating interventions targeting the ongoing inflammatory and metabolic dysfunction characteristic of non-resolving colitis, a clinically relevant phase for therapeutic development. However, we emphasize that this is a preclinical proof-of-concept study, and all findings should be interpreted within the constraints of the model system employed.

Multidimensional data—including physiological assessments, histopathology, serum and tissue biochemical markers, transcriptomic profiling, and qPCR validation—demonstrated that DSS-induced inflammation downregulates genes associated with mitochondrial oxidative phosphorylation in skeletal muscle. In this context, resistance training significantly upregulated mitochondrial gene expression and reduced circulating proinflammatory cytokines (TNF-α and IL-6), consistent with an improvement in muscle metabolic state and attenuation of systemic inflammation, thereby potentially creating a more favorable physiological context for anti-TNF-α therapy.

Mitochondrial dysfunction is a hallmark feature of IBD, characterized by increased oxidative stress and impaired ATP production [38]. In the present study, DSS-induced inflammation led to downregulation of genes related to mitochondrial oxidative phosphorylation in skeletal muscle, including ND1, ND4, and COX1, suggesting that systemic inflammation can negatively impact muscle energy metabolism. These observations are consistent with previous reports linking IBD-associated sarcopenia to mitochondrial impairment [39]. Evidence from both clinical samples and animal models indicates reduced mitochondrial electron transport chain activity and decreased tricarboxylic acid (TCA) cycle metabolites in IBD, reflecting widespread energy metabolism disturbances [40,41]. Additionally, molecular regulators such as noncoding RNAs (e.g., GATA6-AS1) may participate in the modulation of mitochondrial function. Collectively, mitochondrial dysfunction represents a critical factor underlying IBD-associated muscle loss and systemic metabolic dysregulation, highlighting the importance of maintaining skeletal muscle mitochondrial integrity in this context [42,43,44].

In this study, resistance training upregulated the expression of genes associated with mitochondrial oxidative phosphorylation in skeletal muscle and was associated with modulation of upstream pathways—such as PPAR signaling, tyrosine metabolism, and retinoid (vitamin A/ATRA) pathways—that collectively may contribute to reshaping muscle metabolic state. These findings are consistent with prior reports that resistance exercise promotes mitochondrial adaptations and regulates energy metabolism [45,46]. Mechanistically, resistance training is known to increase PGC-1α expression, which in turn drives mitochondrial biogenesis and augments oxidative capacity. PPARs, as nuclear receptor transcription factors, play pivotal roles in skeletal muscle fatty acid oxidation, energy homeostasis, and inflammation. By upregulating PGC-1α, resistance training may enhance PPAR-mediated lipid metabolism and mitochondrial biogenesis, thereby strengthening oxidative phosphorylation gene expression and reducing accumulation of pro-inflammatory metabolic byproducts. However, the present study did not directly measure PGC-1α expression, PPAR activity, or mitochondrial function at the protein or functional level, and these mechanistic interpretations remain hypothetical.

In this study, DSS-induced colitis mice exhibited pronounced systemic inflammation, characterized by elevated serum IL-6 and TNF-α levels, as well as marked upregulation of inflammatory mediators such as IFN-γ and IL-1β in skeletal muscle. Resistance training partially suppressed IL-1β and IL-6 expression but had limited effects on canonical proinflammatory pathways, including TNF, IL-17, and NF-κB. In contrast, infliximab, as an anti-TNF biologic, effectively blocked TNF-mediated inflammatory signaling and significantly reduced IFN-γ, TNF-α, and MMP9 expression, demonstrating broad-spectrum anti-inflammatory effects. Importantly, the combination of resistance training and anti-TNF therapy produced a significant additive effect in suppressing inflammatory mediators. These findings suggest a potential strategy to enhance therapeutic responsiveness in patients with suboptimal anti-TNF treatment, although direct clinical evidence is lacking and further investigation is required.

This study has several limitations that should be considered when interpreting the findings. First, the DSS-induced colitis model employed a single 10-day DSS cycle followed by an 8-week recovery phase, which is more accurately characterized as a subacute model with prolonged systemic consequences. This model primarily recapitulates epithelial barrier disruption and innate immune activation and does not replicate the complex adaptive immune mechanisms that underlie anti-TNF therapy failure in human IBD, including T-cell and B-cell-mediated pathways, immunogenicity, or stromal resistance driven by OSM/OSMR signaling. Consequently, our findings should not be interpreted as demonstrating that resistance training overcomes biologic resistance; rather, they indicate that resistance training provides complementary anti-inflammatory and metabolic benefits under anti-TNF-α exposure in this colitis model. Second, the sample size was relatively small. This was an exploratory preclinical study, and formal a priori power calculations were not performed because effect sizes for the combined intervention were unknown. This limited sample size may constrain the detection of modest effect sizes, particularly for interaction effects, and precludes definitive subgroup analyses. Third, this study was conducted exclusively in male mice. Given that IBD affects both sexes and sex-based differences in immune regulation and muscle physiology are well documented, the generalizability of our findings to females remains unknown. Fourth, the representative histological images presented may not fully capture the graded differences in inflammatory severity across groups. Although blinded semi-quantitative scoring was employed, quantitative morphometric analyses and immunohistochemical quantification of inflammatory infiltrates were not performed. Fifth, mechanistic interpretations remain hypothetical. While we propose a “muscle–immune–gut” axis as a conceptual framework, no direct mechanistic experiments were performed to validate this hypothesis, and mitochondrial outcomes were assessed solely through gene expression profiling without accompanying functional assays. Finally, the anti-TNF-α approach employed the human chimeric antibody infliximab. Although anti-TNF-α therapy has been widely used in murine colitis models and produced biologically meaningful effects, its affinity for murine TNF-α is lower than that for human TNF-α, and some observed effects may involve Fc-mediated mechanisms or underestimate the true impact of complete TNF-α neutralization.

## 5. Conclusions

**Figure 6:**
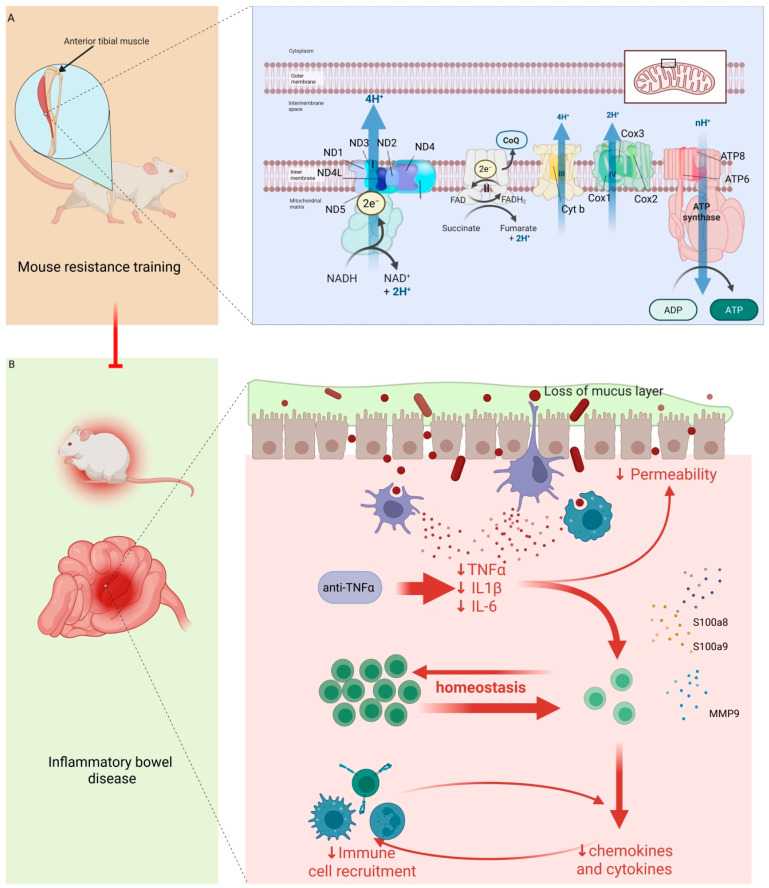
A proposed mechanism by which resistance training increases muscular oxidative phosphorylation gene expression and reduces systemic inflammation to potentiate anti-TNF therapy in IBD. (**A**) Resistance training enhances mitochondrial biogenesis and oxidative phosphorylation gene expression in skeletal muscle, increasing ATP production efficiency. (**B**) Concurrently, it suppresses macrophage activation and systemic proinflammatory cytokine levels. These combined effects lead to profound inhibition of mucosal inflammation and tissue remodeling pathways in the gut, restoration of muscle oxidative phosphorylation gene expression and physical performance, and overall improvement in IBD pathology. When paired with TNF-α blockade, this integrated approach significantly increases biologic treatment responsiveness and ameliorates the disease course. Created in BioRender. Xu, P. (2026) https://BioRender.com/jfd9svi (accessed on 31 January 2026).

This preclinical study provides initial evidence that resistance training is associated with improved muscle mitochondrial gene expression, attenuated systemic inflammation, and enhanced outcomes of anti-TNF-α therapy in a DSS-induced colitis mouse model (Figure 6). The observed effects were largely independent and additive. These findings suggest that muscle-targeted interventions may create a more favorable inflammatory milieu for biologic therapy, but they do not establish causality or clinical efficacy. The proposed “muscle–immune–gut” axis remains a conceptual framework requiring direct experimental validation. Future studies should validate these findings in anti-TNF resistance models, include both sexes with larger sample sizes, incorporate direct mitochondrial functional assays and quantitative histomorphometry, investigate mechanisms through tissue-specific models and secretome profiling, and ultimately evaluate the feasibility and safety of combined exercise and biologic therapy in IBD patients.

## Figures and Tables

**Table 1:** DAI scoring table.

Parameter	Score	Criteria
Body Weight Loss	0	None
	1	1–5%
	2	5–10%
	3	10–20%
	4	>20%
Stool Consistency	0	Normal formed pellets
	2	Loose, mucus-like stool
	4	Diarrhea
Fecal Bleeding	0	No blood
	1	Occult blood positive
	2	Occult blood positive, visible spots
	4	Gross bleeding (visible blood)

## Data Availability

The datasets presented in this study can be found in online repositories (BioProject ID: PRJNA1366076), accessible at https://www.ncbi.nlm.nih.gov/bioproject/PRJNA1366076 (accessed on 31 January 2026). Further inquiries can be directed to the corresponding author.

## Abbreviations

The following abbreviations are used in this manuscript:

IBD	inflammatory bowel disease
anti-TNF-α	anti-tumor necrosis factor-alpha
DSS	dextran sulfate sodium
UC	ulcerative colitis
IFX	infliximab
CD	Crohn’s disease
OSM	oncostatin M
DAI	disease activity index
OD	optical density
DEGs	differentially expressed genes
GO	Gene Ontology
KEGG	Kyoto Encyclopedia of Genes and Genomes
ANOVA	analysis of variance
ECM	extracellular matrix
CAMs	cell adhesion molecules

## Appendix A

**Table A1:** Histological Scoring Criteria for DSS-Induced Colitis.

Parameter	Score	Histological Features
Inflammatory Cell Infiltration	0	No increase in inflammatory cells; normal mucosa.
	1	Mild increase in inflammatory cells in the lamina propria.
	2	Moderate increase in inflammatory cells with focal extension into the submucosa.
	3	Marked increase in inflammatory cells with diffuse infiltration into the submucosa and muscularis layers.
	4	Severe transmural inflammatory infiltrates with lymphoid aggregates and/or crypt abscess formation.
Crypt Architecture Disruption	0	Normal crypt structure; regular spacing and intact epithelium.
	1	Mild crypt irregularity with focal epithelial cell loss.
	2	Moderate crypt distortion with partial crypt loss (<30% of crypts affected).
	3	Severe crypt distortion with extensive crypt loss (30–70% of crypts affected) and branching.
	4	Complete crypt obliteration (>70% crypt loss) with epithelial denudation.
Mucosal Ulceration/Erosion	0	Intact mucosal surface; no erosions or ulcerations.
	1	Focal superficial epithelial erosion involving <10% of the mucosal surface.
	2	Multifocal erosions involving 10–30% of the mucosal surface.
	3	Extensive ulceration involving 30–50% of the mucosal surface with granulation tissue formation.
	4	Widespread ulceration involving >50% of the mucosal surface with deep ulceration extending into the submucosa.

Total Histological Score = Sum of scores for Inflammatory Cell Infiltration + Crypt Architecture Disruption + Mucosal Ulceration/Erosion (Range: 0–12); Scoring Procedure: All histological sections were coded and evaluated independently by two trained investigators blinded to the experimental group allocation.

**Table A2:** Primer pairs and sequences used in this study.

Classification	Name	Forward	Reverse
Related to oxidative phosphorylation	ND1	GGCTATATACAACTACGCAAAGGC	GGTAGATGTGGCGGGTTTTAGG
	ND2	CTTCTGAGTCCCAGAGGTTACC	GAGAGTGAGGAGAAGGCTTACG
	ND3	CTGATGGACTCTCAACGCTGCA	CTCAGAGCGTGATGCCTGGCT
	ND4	CCCTCGTAGTAACAGCCATTCTC	CGACTGTGAGTGCGTTCGTAGT
	ND4L	CCTGGATGATAGAACCTAACGGG	CTCTCCAGCGAAGGTGTAGACA
	ND5	GTCGAGCATCAAGAAAACCGTCC	GCGGTCAGTAATCCTCAGAGGA
	ND6	GCGATGGCTATTGAGGAGTATCC	CACAGCACCAATCCTACCTCCA
	cyt b	TCCACGTTTGGTCACTGCCGAT	GAAGAGGCGAAATGTGGCAGAC
	COX1	GAATGCCACCTTCATCCGAGAAG	GCTCACATTGGAGAAGGACTCC
	COX2	GCGACATACTCAAGCAGGAGCA	AGTGGTAACCGCTCAGGTGTTG
Related to inflammatory responses	IL6	TACCACTTCACAAGTCGGAGGC	CTGCAAGTGCATCATCGTTGTTC
	IL1β	TGGACCTTCCAGGATGAGGACA	GTTCATCTCGGAGCCTGTAGTG
	TNF-α	GGTGCCTATGTCTCAGCCTCTT	GCCATAGAACTGATGAGAGGGAG
	IFN-γ	CAGCAACAGCAAGGCGAAAAAGG	TTTCCGCTTCCTGAGGCTGGAT
	S100a8	CAAGGAAATCACCATGCCCTCTA	ACCATCGCAAGGAACTCCTCGA
	S100a9	TGGTGGAAGCACAGTTGGCAAC	CAGCATCATACACTCCTCAAAGC
	MMP9	GCTGACTACGATAAGGACGGCA	TAGTGGTGCAGGCAGAGTAGGA

**Table A3:** Multiple comparative tests of body weight and DAI of mice in each group.

Šídák’s Multiple Comparisons Test	Mean Difference	95.00% CI for Difference	Statistic	Adjusted *p* Value
Body Weight				
Week 4				
Control vs. DSS	1.562	0.040 to 3.084	3.81	0.043
Week 5				
Control vs. DSS	2.345	0.567 to 4.123	4.94	0.010
Control vs. DSS + anti-TNF-α	1.697	0.483 to 2.910	5.01	0.006
Week 6				
Control vs. DSS	2.502	0.788 to 4.216	5.87	0.006
Control vs. DSS + Training	2.177	0.429 to 3.924	5.33	0.017
Control vs. DSS + anti-TNF-α	2.057	0.301 to 3.813	4.29	0.020
Control vs. DSS + Training + anti-TNF-α	1.725	0.010 to 3.440	3.81	0.048
Week 7				
Control vs. DSS	2.932	1.617 to 4.246	8.56	<0.001
Control vs. DSS + Training	2.153	0.836 to 3.471	6.58	<0.001
Control vs. DSS + anti-TNF-α	2.052	0.734 to 3.370	5.90	<0.001
Control vs. DSS + Training + anti-TNF-α	1.412	0.070 to 2.753	3.87	0.038
DSS vs. DSS + Training	−0.778	−1.553 to −0.004	3.63	0.049
DSS vs. DSS + anti-TNF-α	−0.880	−1.753 to −0.007	3.60	0.048
DSS vs. DSS + Training + anti-TNF-α	−1.520	−2.488 to −0.552	5.67	0.003
Week 8				
Control vs. DSS	3.285	1.725 to 4.845	7.58	<0.001
Control vs. DSS + Training	2.062	0.806 to 3.317	6.00	0.002
Control vs. DSS + anti-TNF-α	2.237	0.984 to 3.490	6.54	0.001
Control vs. DSS + Training + anti-TNF-α	1.472	0.031 to 2.912	3.65	0.044
DSS vs. DSS + Training + anti-TNF-α	−1.813	−3.403 to −0.223	4.09	0.023
DAI				
Week 1				
Control vs. DSS	−1.833	−2.625 to −1.042	11	0.001
Control vs. DSS + Training	−1.000	---		<0.001
Control vs. DSS + anti-TNF-α	−1.167	−1.958 to −0.375	7	0.009
Control vs. DSS + Training + anti-TNF-α	−1.000	---		<0.001
DSS vs. DSS + Training	0.833	0.042 to 1.625	5	0.040
Week 2				
Control vs. DSS	−2.833	−3.625 to −2.042	17	<0.001
Control vs. DSS + Training	−1.833	−2.625 to −1.042	11	0.001
Control vs. DSS + anti-TNF-α	−2.000	---		<0.001
Control vs. DSS + Training + anti-TNF-α	−1.333	−2.334 to −0.333	6.33	0.015
DSS vs. DSS + anti-TNF-α	0.833	0.042 to 1.625	5	0.040
Week 3				
Control vs. DSS	−3.000	−4.226 to −1.774	11.62	<0.001
Control vs. DSS + Training	−1.833	−2.625 to −1.042	11	0.001
Control vs. DSS + anti-TNF-α	−2.333	−3.334 to −1.332	11.07	0.001
Control vs. DSS + Training + anti-TNF-α	−1.333	−2.334 to −0.333	6.33	0.015
DSS vs. DSS + Training + anti-TNF-α	1.667	0.084 to 3.249	5	0.040
Week 4				
Control vs. DSS	−2.833	−3.625 to −2.042	17	<0.001
Control vs. DSS + Training	−2.000	---		<0.001
Control vs. DSS + anti-TNF-α	−2.333	−3.334 to −1.332	11.07	0.001
Control vs. DSS + Training + anti-TNF-α	−1.000	---		<0.001
DSS vs. DSS + Training	0.833	0.042 to 1.625	5	0.040
DSS vs DSS + Training + anti-TNF-α	1.833	1.042 to 2.625	11	0.001
DSS + Training vs. DSS + Training + anti-TNF-α	1.000	---		<0.001
DSS + anti-TNF-α vs. DSS + Training + anti-TNF-α	1.333	0.333 to 2.334	6.33	0.015
Week 5				
Control vs. DSS	−2.667	−3.668 to −1.666	12.65	<0.001
Control vs. DSS + Training	−1.667	−3.249 to −0.084	5	0.040
Control vs. DSS + anti-TNF-α	−2.167	−3.626 to −0.708	7.05	0.009
DSS vs. DSS + Training + anti-TNF-α	2.000	0.267 to 3.734	5.48	0.027
Week 6				
Control vs. DSS	−2.333	−3.916 to −0.751	7	0.009
Control vs. DSS + anti-TNF-α	−1.833	−2.625 to −1.042	11	0.001
DSS vs. DSS + Training + anti-TNF-α	2.000	0.774 to 3.226	7.75	0.006
Week 7				
Control vs. DSS	−2.333	−3.916 to −0.751	7	0.009
Control vs. DSS + anti-TNF-α	−1.833	−2.625 to −1.042	11	0.001
DSS vs. DSS + Training + anti-TNF-α	2.000	0.774 to 3.226	7.75	0.006
Week 8				
Control vs. DSS	−2.500	−3.562 to −1.438	11.18	0.001
Control vs. DSS + anti-TNF-α	−1.833	−3.292 to −0.374	5.97	0.019
DSS vs. DSS + Training + anti-TNF-α	2.000	0.774 to 3.226	7.75	0.006

**Table A4:** Quadriceps muscle weight and colon length across experimental groups.

Group	Quadriceps Muscle Weight (mg)	Colon Length (cm)
Control	177.5 ± 8.84	8.93 ± 0.65
DSS	124.33 ± 8.71 ***	5.78 ± 0.47 ***
DSS + Training	148.67 ± 6.25 ^†††^	6.98 ± 0.31 ^††^
DSS + anti-TNF-α	140.17 ± 4.26 ^††^	7.25 ± 0.38 ^†††^
DSS + Training + anti-TNF-α	166.67 ± 4.37 ^†††^	8.10 ± 0.35 ^†††^

Data are presented as mean ± SD (*n* = 6 per group). *** *p* < 0.001 versus Control; †† *p* < 0.01, ††† *p* < 0.001 versus DSS by Bonferroni post hoc test.

**Table A5:** F Values for Two-Way ANOVA.

Parameter	Control + No Anti-TNF-α	Control + Anti-TNF-α	Training + No Anti-TNF-α	Training + Anti-TNF-α	Main Effect of Anti-TNF-α	Main Effect of Training	Interaction (Anti-TNF-α× Training)	*p*	eta	Confidence Interval of Interaction
Grip Strength test	46.67 ± 2.50	64.67 ± 3.50	53.50 ± 3.99	74.00 ± 3.95	177.75	31.34	0.75	0.397	0.036	−3.70–8.70
Tensile test	2.28 ± 0.14	2.74 ± 0.21	3.21 ± 0.11	3.48 ± 0.17	29.10	158.01	2.12	0.161	0.096	−0.54–0.15
Swimming time	197.17 ± 9.24	242.17 ± 9.24	275.17 ± 9.24	299.17 ± 9.24	83.66	320.24	7.75	0.011	0.279	−21.00 –21.00
TNF-α	306.31 ± 18.16	266.58 ± 12.25	222.60 ± 16.06	182.61 ± 9.93	45.58	201.67	0.10	0.983	0.001	−24.38–24.89
IL-6	542.07 ± 56.19	425.83 ± 25.19	324.86 ± 31.49	243.87 ± 29.02	41.49	169.92	1.33	0.263	0.062	−99.13–28.62
LDH	949.64 ± 26.95	824.47 ± 48.19	765.84 ± 21.59	643.59 ± 27.24	86.30	187.48	0.001	0.914	0.001	−52.88–47.04
CK	548.83 ± 33.36	434.11 ± 32.73	420.23 ± 11.39	375.87 ± 35.13	42.79	59.03	8.37	0.009	0.295	−148.90–8.15

**Table A6:** One-way ANOVA of serum TNF-α, IL-6, LDH, and CK levels among experimental groups.

Variable	Blank Group	Control + No Anti-TNF-α	Control + Anti-TNF-α	Training + No Anti-TNF-α	Training + Anti-TNF-α	F	*p*
TNF-α	144.62 ± 12.03	306.31 ± 18.16	266.57 ± 12.25	222.60 ± 16.06	182.61 ± 9.93	126.89	<0.001
IL-6	108.40 ± 18.09	542.07 ± 56.19	425.83 ± 25.19	324.86 ± 31.49	243.87 ± 29.02	139.46	<0.001
LDH	517.56 ± 47.47	949.64 ± 26.95	824.47 ± 48.19	765.84 ± 21.59	643.59 ± 27.24	127.35	<0.001
CK	339.06 ± 21.39	548.83 ± 33.36	434.11 ± 32.73	420.23 ± 11.39	375.87 ± 35.13	47.24	<0.001

**Table A7:** Blinded semi-quantitative histological scoring of colonic tissue across experimental groups.

Group	Mean Rank	Median (IQR)
Control	3.58	1.00 (0.00, 1.00)
DSS	27.5	9.50 (9.00, 10.00) ***
DSS + Training	19.67	7.00 (6.00, 8.00)
DSS + anti-TNF-α	17.33	6.00 (6.00, 7.00)
DSS + Training + anti-TNF-α	9.42	3.50 (3.00, 4.00) ^††^

Data are presented as mean rank and median (IQR). *n* = 6 per group. Kruskal–Wallis test: χ^2^ = 26.839, df = 4, *p* < 0.001. *** *p* < 0.001 versus Control; ^††^ *p* < 0.01 versus DSS by Dunn’s post hoc test with Bonferroni correction.
